# Knowledge, attitudes and practices of health professionals and women towards medication use in breastfeeding: A review

**DOI:** 10.1186/1746-4358-6-11

**Published:** 2011-08-26

**Authors:** Safeera Y Hussainy, Narmin Dermele

**Affiliations:** 1 Department of Pharmacy Practice, Centre for Medicine Use and Safety, Faculty of Pharmacy and Pharmaceutical Sciences, Monash University, Australia

## Abstract

Many breastfeeding women require and regularly take medicines, especially those available over-the-counter, and the safe use of these is dependent on the advice provided by health professionals such as general practitioners and pharmacists. The primary aim of this review therefore, was to investigate the literature relating to health professionals' and women's knowledge, attitudes and practices towards medication use and safety in breastfeeding. The limited literature that was uncovered identified that general practitioners and pharmacists have poor knowledge, but positive attitudes, and variable practices that are mostly guided by personal experience. They tend to make decisions about the use of a medicine whilst breastfeeding based on the potential 'risk' that it poses to the infant in terms of possible adverse reactions, rather than its 'compatibility' with breast milk. The decision-making process between health professionals and women is usually not a negotiated process, and women are often asked to stop breastfeeding whilst taking a medicine. Women, in turn, are left dissatisfied with the advice received, many choosing not to initiate therapy or not to continue breastfeeding. Some directions for future research have been suggested to address the issues identified in this critical area. This review is important from a societal perspective because many breastfeeding women require and regularly take medications, especially those available without prescription, and the safe use of these is dependent on the advice provided by health professionals, which is ultimately influenced by their knowledge, attitudes and practices. However, there is an absence of high quality evidence from randomised controlled trials on the safety of medications taken during breastfeeding, which naturally would hinder health professionals from appropriately advising women. It is equally important to know about women's experiences of advice received from health professionals, and whether there is consistency between recommendations made across resources on medication safety in breastfeeding, in order to gain a full understanding of the issues prevalent in this area of practice.

## Review

In July-October 2010, keywords (e.g. health professionals, doctors, nurses, pharmacists, lactation, breastfeeding, medication, medicine, knowledge, attitude/s, practice/s, behaviour/s) were used, either separately or in combination, to search databases such as Ovid, Pub Med, International Pharmaceutical Abstracts and Google Scholar. The search was restricted to articles published on primary research data, in English, and from 1990 onwards. The same keywords were used to search relevant journals such as *BMC Women's Health, Medical Journal of Australia, International Breastfeeding Journal *and *Journal of Human Lactation*. The reference lists of relevant articles retrieved from these journals were hand-searched for additional studies.

Thirty-one publications were found that were assessed for relevance to the topic area, 15 of which were not within the scope of this review - 13 that did not investigate health professionals' breastfeeding knowledge, attitudes or practices in the context of prescribing medication for women [1-13]; one that did not determine women's experiences with receiving advice from health professionals on medication use and safety in breastfeeding [14]; and another that was a review of primary research data [15].

Tables 1, 2 and 3 show the 17 publications that were critiqued [16-32]. These reported on studies that mostly used cross-sectional designs; generally had low response rates (RRs); and were undertaken in Australia, Canada, Israel, The Netherlands, USA and United Kingdom (UK), and whose findings therefore cannot be generalised to other settings.

**Table 1:** Publications (in chronological order) on health professionals' knowledge, attitudes and practices towards medication use and safety in breastfeeding

Publication details and country	Study design, participants and setting	Study aims	Key findings
[25] Merlob P, Bracha S, Kaplan B. Drug use in pregnancy and breast feeding: the role of the pharmacist. The International Journal of Risk and Safety in Medicine 1998, 11: 45-7. Israel.	66 pharmacists working at Rabin Medical Center and from private pharmacies were interviewed (via telephone or personal visit). No details were provided on how participants were selected for the study and how their details were obtained.	To examine the rate of pharmacist counselling of women who use medications during pregnancy and/or breastfeeding.	Only 9% (6/66) of pharmacists said they made a practice of asking women two basic questions: "Are you pregnant or nursing?" and "Did you receive an explanation (of the drug(s) in question) from your physician? In no case did the pharmacist offer information at their own initiative.
[22] Jones W, Brown D.^‡ ^The pharmacist's contribution to primary care support for lactating mothers requiring medication. Journal of Social and Administrative Pharmacy 2000, 17: 88-98. England.	Mail questionnaire sent to 939 GPs and 948 community pharmacists in the South and West NHS Executive region of Hampshire, Wiltshire, Dorset and the Isle of Wight. GPs were selected by systematically sampling every fifth name from lists supplied by the local health commission, and pharmacists were identified from information supplied by the Royal Pharmaceutical Society of Great Britain.	To investigate the knowledge and attitudes of GPs and community pharmacists to the safety of infants exposed to medications via breast milk and the importance of continuing breastfeeding.	RR (GPs) = 63% (590/939) and RR (pharmacists) = 68% (641/948). 80% (473/590) of GPs had personal/partner experience of breastfeeding, with the majority (83%, 490/590) agreeing/strongly agreeing with the statement that "Breastfeeding is a health promotion issue". Most pharmacists (82%, 523/641) also agreed/strongly agreed with this statement and many (66%, 422/641) had personal/partner experience of breastfeeding. The majority of GPs (93%, 551/590) said that they would always ask a woman with a baby how she was feeding her child before prescribing medications for her, with significantly fewer pharmacists (318/641, 50%) doing the same. Only 22% (129/590) of GPs said that they would always ask this question to a woman with a toddler. The importance of the breastfeeding relationship for women beyond 6 months was similarly underestimated by pharmacists. Most pharmacists (76%, 486/641) also said that GPs never contacted them for information on medication use and safety in breastfeeding, in contrast to some GPs who said that they would contact the pharmacist (15%), a drug information centre (9%) or the manufacturer (7%). GPs felt it was quite important to inform a woman of potential side effects of a medication if she continues to breastfeed whilst taking it, and they often did this. Pharmacists, on the other hand, while feeling that it was very important to inform a breastfeeding woman, didn't often do so, despite the fact that many reported being asked by women about medication safety (pertaining to, e.g. tranqulisers, hypnotics, oral contraceptives, cough/cold remedies, laxatives) from once a week (46%) to once a month (40%). 9 pharmacists stated that it was the woman's responsibility to inform them that she was breastfeeding and to initiate the query. 50% of GPs and 43% of pharmacists rated the quality of information available on medication use and safety in breastfeeding as acceptable, compared to 22% of GPs and 28% of pharmacists who thought it was of insufficient quality. Interprofessional workshops on the topic were appreciated more by pharmacists (72%, 459/641) than GPs (43%, 255/590). Pharmacists were also found to be more interested (83%) in undertaking a distance learning package in this area than GPs (45%).
[30] Lee A, Moretti ME, Collantes A, Chong, D, Mazzotta P, Koren G, Merchant SS, Ito S. Choice of breastfeeding and physicians' advice: A cohort study of women receiving propylthiouracil. Pediatrics 2000, 106(1):27-30. Canada.	Mail questionnaire sent in 1997 to all Ontario endocrinologists (92) and randomly selected family physicians (300) listed in the *Canadian Medical Directory*. Two case vignettes were presented in the survey - 1) a breastfeeding woman requires 300 mg/day of propylthiouracil (PTU) and has no preference for a feeding method; and 2) a woman on the same dose wishes to breastfeed. Participants were asked to state if they would recommend breastfeeding, and whether they had had to provide such advice in the past year. If the respondent was unsure of their recommendation, they were asked to select from a list which information sources they would consult to obtain an answer.	To examine physicians' attitude toward PTU therapy during breastfeeding.	RR = 72% (endocrinologists) and 38% (family physicians). 47% of endocrinologists, compared with only 1% of family physicians, had given advice within the last year on the use and safety of PTU during breastfeeding. Of all physicians who indicated that they would not recommend breastfeeding in case 1, 2 or both, 81% listed drug amount found in the breast milk as a primary reason for not recommending breastfeeding. 8 family physicians and 5 endocrinologists who were unsure of the compatibility of PTU with breastfeeding provided information sources they would consult. Reference books (66%) were favoured amongst family physicians, followed by colleagues (60%) and other sources (57.5%). Literature searches were chosen by all endocrinologists, followed by reference books and colleagues with or without an additional source. 1 in 4 endocrinologists who were found to be against breastfeeding during PTU treatment reported that they would change their advice to being in favour of breastfeeding if the woman wanted to breastfeed.
[28] Schrempp S, Ryan-Haddad A, Gait KA. Pharmacist counseling of pregnant or lactating women. J Am Pharm Assoc (Wash) 2001, 41: 887-90. USA.	Mail questionnaire sent in 1998-99 to 265 pharmacists practicing in community pharmacies in Nebraska, with adequate representation of rural pharmacists by using a stratified sampling technique. No details were provided on how potential participants' details were obtained. Repeat mailing was done to all non-respondents using a modified Dillman technique.	To provide a better understanding of the role pharmacists play in counselling pregnant or breastfeeding women and to assess pharmacists' comfort level with counselling these patients.	RR = 42% (110/265). Respondents reported counselling pregnant/breastfeeding women an average of 2.8 times per week. Pharmacists rated themselves as both qualified to make, and comfortable with making, therapeutic recommendations for this patient group. Compared with pharmacists who had practiced for *greater *than 30 years, those who had practiced for *less *than 30 years felt more qualified and comfortable, and believed that pharmacy schools did not provide adequate training, and therefore placed greater value on continuing education, in this area. Pharmacists varied in their responses to whether they would recommend medications for 7 common OTC-treatable conditions (cough, cold, analgesia, constipation, diarrhoea, insomnia and heartburn) e.g. for cough, 55% said they wouldn't recommend any medications compared with only 38% who said the same for conditions requiring analgesia. Some pharmacists also recommended some medications that are unsafe in breastfeeding (e.g. 10% recommended diphenhyramine for insomnia and 16% recommended dextrometorphan for cough). Several commented about alternative, non-pharmacological treatments they would prefer to recommend first for 6/7 of the conditions.
[23] Jones W, Brown D.^‡ ^The medication vs breastfeeding dilemma. Br J Midwifery 2003, 11(9): 550-55. England.	See publication 22 in this table.	As for publication 22 in this table.	The figures reported in this publication differ slightly from those in publication 22, despite being the same study, and this publication reports less than, but similar results to, publication 22. Therefore, only new results are presented here. 80% of GPs had personal/partner experience of breastfeeding, and comments made by participants indicated that their experiences were different to those of the pharmacists. 3% (19/641) of pharmacists said that they were never asked about medication safety in breastfeeding by their customers, compared to 9% (60/641) who reported being asked around once a week. Both GPs (76%, 450/589) and pharmacists (81%, 522/641) thought it was difficult to stop breastfeeding abruptly and made varying comments about providing information to breastfeeding women on medication safety and use.
[31] Long L, Montouris G. Knowledge of women's issues and epilepsy (KOWIE-II): a survey of health care professionals. Epilepsy Behav 2005, 6(1):90-3. USA.	Attendees of the American College of Physicians 2003 annual meeting invited to undertake a web-based version of the KOWIE-II, a validated and reliable tool for investigating issues that affect women with epilepsy, such as effects of antiepileptic medicines on oral contraception, bone health, sexual function, pregnancy and breastfeeding. Participants could choose either 'true', 'false' or 'don't know' to each item.	To assess what physicians know about issues pertaining to women with epilepsy.	RR not calculated. 202 respondents completed the survey, the majority of whom were physicians (92%). 4% were medical students and the remaining 2% were other health professionals not named. Most respondents were male (66%) and their mean age was 38 years. The majority (80%) treated less than 10 patients with epilepsy per month. The most common specialty was internal medicine (74%), followed by pulmonology (14%) and general practice (3%). In response to the item, 'Most women taking antiepileptic drugs can safely breastfeed', only 47% identified the correct answer ('true'), with 22% responding in the negative ('false') and 32% not knowing ('don't know').
[17] Amir LH, Pirotta MV.* Medicines for breastfeeding women: a postal survey of general practitioners in Victoria. Med J Aust 2009, 191:126. Australia.	Mail questionnaire sent in 2007-8 for anonymous completion to 640 GPs who provided shared maternity care at the RWH in Melbourne, Victoria. Repeat mailing was done to all potential participants, and a reminder postcard was sent in-between the first and second mailings. See publication 21 in this table for information on how potential participants were identified in this study.	To describe GPs' current and preferred sources of information about the safety of medications during breastfeeding.	RR = 52% (335/640). Most respondents were women (70%, 233/333), and most (68%, 227/333) had personal/partner experience of breastfeeding for longer than 6 months. 70% of GPs used the internet during consultations and 82% found it to be helpful. Most (73%) obtained information about medications and breastfeeding from their software prescribing program or from dedicated books (61% predominantly used the RWH** reference text, Drugs and breastfeeding), and 51% used telephone advice (predominantly from the RWH pharmacy). However, some GPs wrongly assumed that the drug categories for pregnancy also applied to breastfeeding women. Their software prescribing program and a reliable internet database were preferred by respondents as information sources. The need for an internet database was appealed for using the example of ibuprofen - inconsistent recommendations in manufacturers' product information exist on its safety, and only 31% (102/330) of surveyed GPs were aware that it is safe in breastfeeding even though most (89%, 293/331) were confident about prescribing medications for breastfeeding women.
[32] McAuley JW, Casey J, Long L. An evaluation of pharmacists' knowledge of women's issues in epilepsy. Epilepsy Behav 2009, 14(1):243-6. USA.	A random sample of 500 pharmacists registered in Ohio were sent a mail version of the KOWIE-II questionnaire (see publication × in this table for details of this tool), or completed it prior to a live continuing education seminar. The year of survey distribution was not given and participants' preferences for epilepsy education were not determined either. Data for both methods of administration was pooled and analysed. The percent correct score was calculated per pharmacist.	To assess what pharmacists know about issues pertaining to women with epilepsy.	RR = 22% (109/500, mail survey). 43 surveys were completed at the continuing education seminar. 45% of respondents were female, their mean age was 51 years, and most identified themselves as community pharmacists (79%). Participants' mean number of years spent in their current practice setting was 19 and the average number of patients with epilepsy seen per month was 14. 50% of the respondents treated less than 10 patients with epilepsy per month. In response to the item, 'Most women taking antiepileptic drugs can safely breastfeed', only 34% identified the correct answer, with 36% responding in the negative and 31% not knowing.
[26] Ronai C, Taylor JS, Dugan E and Feller E. The identifying and counseling of breastfeeding women by pharmacists. Breastfeed Med 2009, 4: 91-5. USA.	Written questionnaire administered at pharmacy sites by a student researcher in 2007, to 36/47 pharmacists in Rhode Island (1 questionnaire per pharmacist per site). This was preceded by distribution of the reference text, *Medication and Mothers Milk *(Hale) to pharmacists by the Rhode Island Department of Health in 2006, following preliminary feedback from a number of pharmacists that the book was not available.	To determine what strategies and resources pharmacists were using to identify breastfeeding women and guide medication recommendations.	RR = 92% (33/36). 58% of respondents never asked women if they were breastfeeding and some were concerned that asking this question would cause offense. 61% believed that women should self-disclose to the pharmacist that they are breastfeeding, and one-third thought that the prescribing physician should alert the pharmacy or that they could check by identifying if the woman had received prenatal vitamins. Most pharmacists (85%) reported feeling somewhat or very comfortable giving advice to breastfeeding women. Nearly half (45%) reported receiving enquiries daily or weekly and used various resources to guide their recommendations, most commonly the *Physician's Desk Reference*, the respective pharmacy chain's intranet, and *Medication and Mothers Milk*. All but one pharmacist who had received the latter reference reported using it at least monthly.
[18] Amir LH, Pirotta MV.* Medicines for breastfeeding women: a postal survey of knowledge, attitudes and practices of general practitioners in Victoria, Australia. August 2010, La Trobe University: Melbourne. Australia.	See publication 17 in this table.	To determine GPs' attitudes to breastfeeding; knowledge of one medication that has a side effect of reducing milk supply; advice for breastfeeding women regarding several medications (paracetamol, ibuprofen, metronidazole, St John's wort and lithium); reports of adverse events for infants; and most preferred sources of information about medications for breastfeeding women.	See RR for publication 17. Over half of the GPs were generally supportive of breastfeeding, disagreeing with unsupportive statements e.g. "In most cases a breastfeeding mother must temporarily wean her baby while she is taking prescription medications" (89%, 295/333). 64% (215/335) of respondents could correctly name one medication that had a side effect of reducing milk supply; however, 22% (75) skipped this question. Less than one third knew that ibuprofen (30%, 102/335) and metronidazole (22%, 72/335) are compatible with breastfeeding. Only 2% of GPs (6/333) knew that St John's wort is relatively compatible. Many (51%, 168/330) said they need to look into the use of lithium (the infant needs to be monitored and breastfeeding is usually discontinued) or that they have concerns about its use (42%, 140/330). Most knew (88%, 291/331) that paracetamol is compatible. The majority of respondents (90%) drew on previous clinical experience to make decisions around medication safety in breastfeeding, with few (n = 6) having told women to stop breastfeeding during therapy. Concern about medico-legal issues was common (76%, 250/330 rated it as an 'important/very important' factor in decision-making for medicines for breastfeeding women). Only 18% (61/335) were able to report an adverse event associated with maternal use of medications whilst breastfeeding (where the most commonly reported adverse event was associated with antibiotic use), and none of the events were serious. GPs wanted practical information about medications for breastfeeding women available via their software prescribing program (68%) or a reliable internet database (57%).
[21] Jayawickrama HS, Amir LH, Pirotta MV. * GPs' decision-making when prescribing medicines for breastfeeding women: Content analysis of a survey. BMC Research Notes 2010, 3:82. Australia.	See publication 17 in this table. Additional detail about how participants were identified was given in this publication: a current list of 666 GPs was obtained from the RWH. However, details on how 640 of them were selected as potential participants were not provided.	Using content analysis, to determine factors governing GPs' decision-making in response to a question about prescribing medications for a breastfeeding woman.	See RR for publication 17. The most common conditions for which GPs had last had to decide about medication use for a breastfeeding woman were mastitis (24%), other infections (24%) and depressive disorders (21%), where the decision to use anti-infectives was reported to be easier than determining antidepressant use. The 6 organising themes that emerged from 253 responses were: "certainty around decision-making", "uncertainty around decision making", "need for drug information to be available, consistent and reliable", "joint decision-making", "the vulnerable third party", and "infant feeding decision". Certainty was associated with positive feelings such as being happy, comfortable and confident, compared to uncertainty that was associated with negative feelings such as concern and doubt. Various sources were accessed by GPs for information on medication safety in lactation, including pharmacists whose opinions sometimes clashed with their own. The need for reliable and consistent information was noted, especially on complementary medications. Involving the mother and other health professionals in a joint decision-making process was felt to be necessary in facilitating a safe outcome for the woman and increasing the chances of her compliance with the recommended/prescribed medication. GPs undertook "benefits versus risk" assessments when considering medication safety, however didn't consider the potential exposure to the infant (the "third party"). Some GPs advised unnecessary cessation of breastfeeding during medication use and others emphasised its continuance. "Complexity of managing risk in prescribing for breastfeeding women" was identified as the global theme.

^‡^These two publications concern the same study and their results are similar, however publication 23 reports less results than publication 22.

**RWH = Royal Women's Hospital

^# ^These three publications report different aspects of the same study.

**Table 2:** Publications (in chronological order) on women's knowledge, attitudes and practices towards medication use and safety in breastfeeding

Publication details and country	Study design, participants and setting	Study aims	Key findings
[24] Matheson I, Kristensen K, Lunde PKM. Drug utilization in breast-feeding women: A survey in Oslo. Eur J Clin Pharmacol 1990, 38: 453-9. Norway.	Questionnaire mailed to random sample of 20% (n = 1131) of women who gave birth in Oslo in 1985. No details were provided on how participants were selected for the study and how their details were obtained; however, readers are referred to a previous publication (not in English) by the authors for a more detailed description of the study.	To investigate the extent of and predictors of medication use in women during the first months after birth, and to identify those symptoms in women that led to medication use during this time. The general attitude to medications was tested by asking about medication taking behaviour when suffering from a strong headache.	RR = 78% (885/1131). Fewer of those women who were breastfeeding at 4 months after birth (n = 645) were using medications than those who had stopped breastfeeding before 4 months (n = 240). The average number of doses was 166 and 307 respectively in that period. The number of doses was significantly associated with the use of oral contraceptive agents (p < 0.005) and young maternal age (p < 0.05). Most commonly reported conditions for which medications had been used in the 4 month period were dyspepsia, haemorrhoids, mastitis, rash/eczema, cracked nipples, headache, allergy and constipation. Analgesics/antipyretics (31%), dermatologicals (19%) and haemorrhoidals (15%) were most commonly taken at least once during the 4 months after birth, and vitamins and iron preparations were taken more frequently by those still breastfeeding. Long-term medication in breastfeeding women included many medications for which there was incomplete or no data about safety at the time of the study (e.g. cromoglycate, topical corticosteroids, salbutamol, insulin and thyroxine). 35% of breastfeeding women smoked daily, as did 65% of those not breastfeeding at 4 months, and there was no difference between breastfeeding and not breastfeeding women with regards to abstinence from alcohol (17 and 16%), but significantly more of those non breastfeeding were abstainers from coffee. 36% of women had less, 33% had similar, and 17% had more doubts about medication use during lactation than in pregnancy. 13% could not assess the perceived risk of medication use during lactation in relation to that in pregnancy. An association between the number of medications per woman and doubts about medication use during breastfeeding was found (p < 0.001).
[20] Ito S, Koren G, Einarson TR. Maternal noncompliance with antibiotics during breastfeeding. Ann Pharmacother 1993, 27: 40-2. Canada.	Prospective cohort study by telephone follow-up of 203 breastfeeding women who were prescribed antibiotics as monotherapy and consulted the Motherisk Program between January 1990 and July 1991. Telephone interviews were conducted by Motherisk counsellors or trained toxicology students. The program was a consultation and follow-up service at a tertiary care, paediatric hospital in Toronto, for healthcare professionals and women with concerns about exposure to medications, chemicals and radiation during pregnancy and breastfeeding.	To determine the incidence of antibiotic prescription failure in breastfeeding women, and to characterise breastfeeding patterns during antibiotic therapy.	62% (125/203) of breastfeeding women who consulted the Motherisk Program for information about the safety of antibiotics during breastfeeding were followed up within 32 weeks. 15% (19/125) of these women did not initiate therapy and Category* A antibiotics (e.g. metronidazole, chloramphenicol, tetracycline, doxycycline) were associated with the highest rate of non-use in this group of women, all of whom continued breastfeeding during the treatment period. 7% of the remaining 106 women who started taking a prescribed antibiotic stopped breastfeeding during therapy. In this group of women, Category A and B (e.g. norfloxacin, ciprofloxacin, spiramycin) antibiotics were associated with a significantly higher incidence of breastfeeding interruption (p < 0.01) than those in Category C (e.g. penicillins and cephalosporins). This means that 21% of women (26/125) eventually avoided exposing their infant to the medication. Despite reassuring advice from the program's staff (based on data from available texts, the recommendations of the American Academy of Pediatrics, case reports, and the authors' own experience), it was estimated that 1 in 5 women did not initiate therapy or did not continue breastfeeding.
[25] Merlob P, Bracha S, Kaplan B. Drug use in pregnancy and breast feeding: the role of the pharmacist. The International Journal of Risk and Safety in Medicine 1998, 11: 45-7 Israel.	204 women interviewed by a neonatologist (specially trained in teratology) at discharge from a maternity ward (assumed to be located at Rabin Medical Center), two to three days after delivery. No details were provided on how participants were selected for the study.	To examine the rate of pharmacist counselling of women who use medications during pregnancy and/or breastfeeding.	63% (129/204) of women reported that they were counselled by their physician, compared with 9% (18/204) who indicated that they were counselled by the pharmacist. 8% (16/204) of women had read the information leaflet accompanying the medication and in many instances, no leaflet was provided at all.
[22] Jones W, Brown D^‡^. The pharmacist's contribution to primary care support for lactating mothers requiring medication. Journal of Social and Administrative Pharmacy 2000, 17: 88-98. England.	Mail questionnaire sent to 967 breastfeeding women in the South and West NHS Executive region of Hampshire, Wiltshire, Dorset and the Isle of Wight. The women were not a random sample of the study area population as they were systematically selected from the National Childbirth Trust and on a proportional basis to represent all of its branches across the geographical area.	To assess what information mothers were given or wished to be given on medication safety and whether professionals provided this.	RR = 85% (820/967 women). 57% of women reported taking medication after birth, but just over half of the women (51%) recollected being asked if they were breastfeeding. 42% did not recall receiving any medication after birth. The majority of women (279) who were prescribed medication (mostly analgesics and oral contraceptives) recalled being asked by their GP if they were breastfeeding, 29% (131) stated they were not asked, and 30 did not remind their GP that they were breastfeeding. 54% of women said they had bought OTC medications from a pharmacy but only 11% recalled being asked if they were breastfeeding, and of the 82% who said they weren't asked, 34% reminded the pharmacist that they were breastfeeding. 28% were satisfied with the advice given by GPs and pharmacists and 31% felt dissatisfied with the quality of information given. 6% said that they received conflicting advice. 48 women were advised to discontinue breastfeeding, 28 refused the medication, 25 requested an alternative medication, 24 expressed breast milk temporarily, 17 discontinued breastfeeding and 10 sought a second opinion.
[23] Jones W, Brown D^‡^. The medication vs breastfeeding dilemma. Br J Midwifery 2003, 11(9): 550-55. England.	See publication 22 in this table.	As for publication 22 in this table.	The figures reported in this publication differ slightly from those in publication 22, despite being the same study. Therefore, only new results are presented here. 48% (395/820) of women continued to breastfeed at 6 months and 19% (153/820) were breastfeeding at the time of completion of the questionnaire. While 250 of the medications taken by women after birth were analgesics and 63 were antibiotics, a wide range of other medication was given, such as antiepileptics, antihypertensives, antihistamines and thyroxine. Similarly, the majority of prescriptions received from GPs once women were home were for antibiotics and analgesics, but also included antidepressants and iron supplements.
[30] Lee A, Moretti ME, Collantes A, Chong, D, Mazzotta P, Koren G, Merchant SS, Ito S. Choice of breastfeeding and physicians' advice: A cohort study of women receiving propylthiouracil. Pediatrics 2000, 106(1):27-30. Canada.	Prospective cohort, observational study. 78 women who contacted the Motherisk program between 1990-97 to enquire about foetal safety of PTU therapy were interviewed postpartum regarding their choice of infant feeding method and relevant advice received from physicians. Women who rang the program to determine its safety in breastfeeding were excluded from the study. Data for 3 groups of women were compared: group 1) women who required PTU postpartum; group 2) women who no longer required the medicine; and group 3) a control group of age-matched women who contacted the program within the same year as their counterparts and who were not taking any chronic medicines or teratogenic or toxic substances.	To determine whether there is a relationship between physicians' advice and women's initiation of breastfeeding during PTU therapy, as well as the extent of physician compliance with evidence-based data on the safety of this medicine in breastfeeding.	66/78 women identified had live births and were included in the study. 55% (36/66) of women were in group 1, 46% (30/66) in group 2, and 36 in group 3. Breastfeeding initiation rates for these groups were 44%, 83% and 83% respectively (group 1 vs group 2, p < 0.01; group 1 vs group 3, p < 0.01), however, demographic characteristics of the 3 groups were similar. In group 1, 10/36 women did not seek advice from physicians and the remaining 26 (72%) received advice from 39 physicians. 62% (24/39) of physicians advised women to breastfeed, 33% (13/29) advised not to breastfeed and 5% (2/29) gave equivocal advice. Of 18 women who received advice in favour of breastfeeding by at least 1 physician, 83% (15/18) initiated breastfeeding. Whereas of the 8/24 women who did not receive advice in favour of breastfeeding, none breastfed. 60% (12/20) of women who chose to formula feed indicated physicians' advice against breastfeeding or their concern about the medicine as the main reason for not breastfeeding. 50 women who breastfed received advice from 22 physicians regarding breastfeeding (20 in favour, 1 against and 1 equivocal), and 11 who formula fed received advice from 17 physicians (4 in favour, 12 against and 1 equivocal). Physicians' advice was the only significant predictor of the woman's choice to breastfeed during PTU treatment (relative risk: 5, 95% CI: 1-23).
[27] Schirm E, Schwagermann MP, Tobi H, de Jong-van den Berg LT. Drug use during breastfeeding. A survey from the Netherlands. Eur J Clin Nutr 2004, 58: 386-90. Netherlands.	Questionnaire given to all women (for completion at home) with a child not older than 6 months, who had visited a Well-Baby Clinic in the province of Friesland. If the woman visited the clinic more than once in the study period, only one questionnaire was handed out, and if someone other than the woman came to the clinic with the child, they were asked to pass on the questionnaire to the woman. This was carried out over a 6-week period in November-December 2002 across 85 Clinics.	To survey medication use by breastfeeding women, and to compare this with non-breastfeeding women. Also to explore whether medication use influenced women's decisions to breastfeed and vice-versa.	RR ≈ 43% (549 returned questionnaires). 82% of respondents breastfed their baby some time during the first 6 months after birth. 66% of all breastfeeding women had used medications, but less frequently than non-breastfeeding women (80%). Vitamins were used more frequently by breastfeeding women, whereas oral contraceptives, iron preparations, and peptic ulcer and psychotropic medications use was higher in non-breastfeeding women. 30% of women hesitated to use medications during breastfeeding, 10% stopped either breastfeeding or medication use to avoid combining the two, 5% took a measure to minimise exposure to the child, and 12% did not breastfeed because of medication use.
[19] Boath E, Bradley E, Henshaw. Women's views of antidepressants in the treatment of postnatal depression. J Psychosom Obstet Gynecol 2004, 25: 221-33. UK.	Questionnaire administered to 35 women, in their homes, who had a baby aged between 6 weeks and 1 year and who either scored above 12 on the Edinburgh Postnatal Depression Scale or were diagnosed with clinical depression by a psychiatrist. Women were participating in a wider study (that the authors have referenced and provides more information on how participants were identified and selected for the study) of the cost-effectiveness of services for PND.	To determine the experiences of postnatally depressed women with regards to antidepressant treatment.	RR = 43% (60/82 women fulfilled the recruitment criteria, however, only 35 were taking antidepressant medication - 5 were on SSRIs**, 25 were prescribed TCAs^#^, 2 of these were subsequently prescribed SSRIs and 3 were subsequently prescribed flupenthixol. One woman was taking flupenthixol alone). All women were of white ethnicity. 13/35 women were breastfeeding and 4/13 (31%) did not want to take the antidepressant for that reason. It was also reported that the literature shows that TCAs and SSRIs are safe for infants, but should be prescribed only after the risk-benefit ratio is clearly outlined and discussed with the woman and her partner.
[29] Turner K, Sharp D, Folkes, Chew-Graham, Women's views of antidepressants as a treatment for postnatal depression: a qualitative study. Fam Pract 2008, 25:450-55. UK.	In-depth interviews between November 2006-June 2007 with 27 women in three UK cities, at their homes or over the telephone. Women had been diagnosed with postnatal depression and taken part in a randomised controlled trial (that the authors referenced and provides more information on how participants were identified and selected for the study), involving antidepressants versus antidepressants and non-directive counselling. A purposeful sampling approach was used to ensure interviews were held with women randomised to different treatment arms and living in different cities.	To determine the experiences and views of postnatally depressed women with regards to antidepressant treatment.	Women expressed concerns about taking antidepressants when breastfeeding, however the associated medications were not reported and comments to illustrate these concerns were also not provided. Moreover, the number of women who were breastfeeding was not given. It is not known whether women specifically discussed with their GP the issue of using antidepressants when breastfeeding. Most women were of white ethnicity (21/27).

*Categories were defined as A = relatively incompatible with breastfeeding; B = probably compatible; and C = safe to use in breastfeeding.

^‡^These two publications concern the same study and their results are similar, however, publication 23 reports less results than publication 22.

**SSRIs = serotonin selective reuptake inhibitors

^#^TCAs = Tricyclic antidepressants

**Table 3:** Publications on recommendations made by resources on medication use and safety in breastfeeding

Publication details and country	Study design and setting	Study aims	Key findings
[16] Akus M, Bartick M. Lactation safety recommendations and reliability compared in 10 medication resources. Ann Pharmacother 2007, 41: 1352-60. USA.	10 frequently used sources of information (see Table 4) by US health professionals on medication safety in lactation were evaluated and compared with each other, for 14 commonly used medications (acyclovir, amlodipine, ampicillin, ciprofloxacin, enalapril, haloperidol, metformin, methotrexate, metoprolol, pantoprazole, paroxetine, sertraline) widely recognised as safe, not safe or neither. The number of medications thought to be safe for each source was also assessed.	To determine the reliability of safety recommendations for medications used during lactation, based on current research and information. Also to determine whether resources may be inappropriately advising the interruption of breastfeeding.	*Medication and Mothers Milk *and *LactMed *had the highest number of safe-rated medications (12/14 and 11.5/14 respectively) among the sources used, compared with the 2 retail pharmacy databases, *Lexi-Comp *and the *Physician's Desk Reference*, which listed the fewest number of safe medications (0.5/14 and 0/14 respectively) and lacked safe listings even for those widely accepted as safe. Of all sources across the sample of 14 medications, *LactMed *more consistently contained more extensive and current citations, suggesting that it may have been the most reliable source at the time of the study. For medications thought to be unsafe, the 2 retail pharmacy databases gave an alternative recommendation 75% of the time.

Table 1 details 11 studies - seven that have been conducted with health professionals only [17,18,21,26,28,31,32] (where references 17-18 and 21 concern the *same *study); two with pharmacists, general practitioners (GPs) and breastfeeding women together [22,23] (these concern the *same *study); another with pharmacists and breastfeeding women [25]; and one with endocrinologists, family physicians and women [30]. In the four studies that involved health professionals and women [22,23,25,30], only the findings on the health professionals have been detailed, and those associated with the women are shown in Table 2.

Table 2 also highlights nine studies - five with breastfeeding women alone [19,20,24,27,29]; two with breastfeeding women, pharmacists and GPs together [22,23] (again, these concern the *same *study); another with breastfeeding women and pharmacists [25]; and one with women, endocrinologists and family physicians [30].

Table 3 includes information on the only study that has been undertaken to determine the recommendations made within resources on medication safety in breastfeeding [16].

A synthesis of the evidence from these studies is presented below. Individual studies are mentioned, where appropriate, to highlight key points or compare and contrast findings between studies.

### Health professionals

No studies were uncovered between 1990-97 on health professionals' knowledge, attitudes and practices towards medication use and safety in lactation. Most of the studies conducted from 1998-2010 are on GPs, also known as family physicians [17,18,21-23,30,31], with no representation from maternal and child health community nurses, who have an important role in this area given that they interact frequently with mothers in the postpartum and postnatal periods, but at the same time do not have prescribing rights or formal knowledge of pharmacology or medications.

While some GPs have been found to be supportive of medication use during breastfeeding [18,21,30], others have unnecessarily advised women to cease therapy whilst breastfeeding [18,21], or to stop breastfeeding temporarily or permanently [22,23] as the risks associated with breastfeeding cessation were viewed to outweigh the benefits and risks of medication use [23]. A high percentage have even advised against breastfeeding initiation [30]. This latter and highly unfavourable situation is occurring even when there is adequate safety data to support the use of several medications whilst breastfeeding, ranging from those used to treat acute and minor ailments (e.g. ibuprofen for pain), to those prescribed for chronic and debilitating conditions (e.g. fluoxetine and sertraline for depression, propylthiouracil [PTU] for hyperthyroidism) [23,30,33].

Indeed, for medications such as antidepressants, the GP's decision to prescribe them should be arrived at by conducting a risk versus benefit assessment [23,33], "taking into account factors such as the maturity of the infant, the frequency of feeds and the volume of milk consumed" [23]. The woman's preferences for treatment must also be considered and are equally important; however, only 4% (23/590) of GPs who responded to the survey by Jones and Brown indicated that the woman would be involved in making an informed choice [22].

In the study by Lee et al, women who received physician advice in favour of breastfeeding were more likely to breastfeed during PTU therapy than formula feed (relative risk: 5, 95% confidence interval: 1-23) [30]. Around 25% of endocrinologists in the same study who responded to case vignettes presented in a postal survey advised against breastfeeding during PTU treatment, but also said they would change their recommendation if the woman expressed the desire to breastfeed. Nevertheless, where a mutual decision to use medication is made, the breastfed infant should be monitored for adverse effects (e.g. sedation, failure to thrive) and if they are suspected, blood samples from the woman and the infant may be taken for laboratory analysis [33].

Some GPs have even been reported to use pregnancy drug categories for breastfeeding [18], which means that their recommendations to either continue or stop breastfeeding may be incorrect. However, GPs (and other health professionals such as pharmacists) have acknowledged that it is difficult to stop breastfeeding abruptly despite their recommendations for women to do so [22,23].

It appears that incorrect recommendations are being made mainly because of poor self-reported [28] or actual [30-32] knowledge, which has been found to be influenced by frequency of contact with women [31,32], as well as health professionals' personal or partner experience of breastfeeding their own children, although the latter may place them in a better position to handle breastfeeding and medication related problems [22,23]. For example, in a mail survey of 265 pharmacists practicing in community pharmacies in Nebraska, United States of America (USA), it was found that for treating insomnia, 10% of the respondents recommended diphenhydramine (contraindicated in lactation), guaiphenesin (no data on safety) and dextromethorphan (might contain ethanol) for breastfeeding women [28]. In other studies, only 34% of pharmacists [32] and 47% of physicians [31] knew that most women taking antiepileptic medicines can safely breastfeed; 66% of pharmacists and 54% of physicians thought they were unsafe or were unsure about their safety.

Similarly, in the study by Schrempp, Ryan-Haddad and Gait, some pharmacists recommended alternative, non pharmacologic treatments first, such as bran cereal or prune juice as natural laxatives for constipation; saline nasal drops, cool mist vaporiser or echinacea for colds; and dietary limitations, including eating smaller meals, for heartburn [28]. While such recommendations are safe, reasonable and are usually trialled before prescribing medications, they work best in conjunction with pharmacological measures and may only provide temporary relief of the breastfeeding woman's symptoms. In addition, pharmacists in this study who had practiced for less than 30 years more highly valued continuing education (CE) on medication safety and use in breastfeeding than pharmacists who had practiced for more than 30 years [28], but were either not asked, or did not indicate, their preference(s) for formats of CE.

Poor knowledge of medication use in breastfeeding may also extend to inadequate medical history-taking skills, where important information is not gleaned from the breastfeeding woman by the health professional. Ronai et al found that 58% of pharmacists surveyed never asked women if they were breastfeeding [26]; however, this study was carried out with a small sample of participants. While Jones and Brown reported that 50% of pharmacists said they would ask a woman with a baby how she was feeding before prescribing an over-the-counter (OTC) medication (available without prescription) for her, many pharmacists stated that it was the responsibility of the woman to inform them that she was breastfeeding when purchasing a medication, with two pharmacists commenting that questions of this nature are too personal and cannot be asked in the pharmacy environment [23].

In the same study, only 22% of GPs surveyed said that they would ask the mother of a 'toddler' (taken to be older than 12 months) if she was breastfeeding before prescribing medication for her [22,23]. This is concerning, given that women may continue to breastfeed after their child turns 12 months old - the minimum age recommended in Australia [34] - meaning that both GPs and pharmacists may not be considering that mothers of children who are no longer newborn may be breastfeeding. Individual comments provided by GPs and pharmacists in the study by Jones and Brown "gave an impression of ambivalence to breastfeeding, particularly as the child got older and that they would advocate artificial milk formula as a substitute" [22].

This attitude, as seen from the woman's perspective, is reflected in the comment below, made by a mother in the study by Jones and Brown [23]. Indeed, the World Health Organization (WHO) and United Nations International Children's Emergency Fund (UNICEF) recommend exclusive breastfeeding for six months, with introduction of nutritionally adequate, safe and appropriate complementary foods (to prevent iron deficiency) and continued breastfeeding thereafter.

*"In my experience, the medical profession do not expect breastfeeding beyond 9 to 12 months. Beyond that they never ask if you are feeding and act surprised when you point it out" *[23] (p. 553).

On the other hand, some GPs practicing in Australia have been reported to feel confident about medication use in breastfeeding women [18]. However, despite their self-reported confidence, in a postal survey of GPs in Victoria, respondents had concerns about medico-legal issues: 76% of GPs rated the possible risk of litigation as "important/very important" [18]. Similarly, it has been found that some pharmacists feel comfortable advising breastfeeding women and are cautious with their recommendations to them [26]. In this study by Ronai et al, pharmacists believed that it is better to stop breastfeeding than risk exposing an infant to a potentially harmful medication [26]. They also commented that there are contradictory and inconsistent resources available on medication use in lactation, as have GPs who have also indicated the need for a reliable internet database (that includes information on complementary medicines), or information via their software prescribing program, [17,18,21] instead of having to rely on pharmaceutical companies for drug information that is usually "over cautious" [21].

In this Victorian survey with GPs, several participants reported that advice their patients had received from community pharmacists sometimes conflicted with their own decisions, unnecessarily resulting in patient alarm [21]. Community pharmacists in these scenarios had questioned GPs' decisions to prescribe fluconazole or metronidazole. It was also found that some GPs were referring to pharmacists at the Royal Women's Hospital (RWH), and held these pharmacists, or others working elsewhere, in high esteem:

*"The pharmacist at RWH *[is] *excellent - gives various sources of information and good opinion re: overall management ..."*[21] (p. 5).

*"Depression. Efexor [venlafaxine]. Checked with Box Hill Hospital pharmacist via phone. Got the most reliable and up to date info ..." *[21] (p. 5).

If pharmacists, particularly those working in community settings, have insufficient knowledge and they are providing information to patients that 'challenge' GPs' decisions, this is a dilemma. If pharmacists continue providing inconsistent information and if their knowledge gaps are not met, they could potentially discredit themselves as medicine experts. Also, as some information provided by pharmacists is based on "personal decision", as demonstrated in the comment below by a participant in the aforementioned study [21], it could potentially be harmful to women.

*"Depression. Most information is 'personal decision' i.e. no good evidence. Reasons for decision - local psychiatrist opinion, RWH pharmacist's opinion ..." *[21] (p. 5).

These findings from the Victorian survey [17,18,21] should be considered in the context of having an unrepresentative sample. The invited sample had more female GPs than males (because more females in Australia are involved in shared maternity care than male GPs), the proportion of female to male respondents is higher than in the invited sample, and those who did respond probably had a greater interest in the topic than non-responders. Taken altogether, it is highly likely that the results in this survey underestimate the true situation.

### Breastfeeding women

No studies were uncovered in 2006 and between 2009-10 on women's knowledge, attitudes and practices towards medication use and safety in lactation. Most of the limited studies undertaken with breastfeeding women from 1990-2008 show that some were inappropriately advised by a health professional to unnecessarily cease or not even commence breastfeeding during the pharmacotherapy [20,22,23,30], and if they hadn't received advice, they still had the same doubts or were more reluctant to take medications during breastfeeding than during pregnancy [24]. Some of these findings, however, may not be relevant anymore as the work was undertaken up to 20 years ago [20,24], and the study with women from Oslo did not have adequate representation, with a lower response rate from women living in high socioeconomic conditions than those from lower socioeconomic environments [24]. Other work [19,29], which includes one study that was recently conducted [29], is also exclusively or mostly limited to women of white ethnicity.

Regardless of these limitations, a proportion of women in some of the studies consequently stopped either breastfeeding or medication use to avoid combining the two [20,22,23,27] or did not breastfeed at all [30], and others took a measure to minimise exposure to the child (e.g. expressing breast milk beforehand, breastfeeding just before medication intake) [27]. However, due to dissatisfaction with the advice they received, some women either did not commence the medication that was prescribed for them or did not stop breastfeeding [20,22,23].

In the study by Jones and Brown, it was found that only 28% of breastfeeding women were satisfied with the advice given by their GP and pharmacist, compared to 31% who were dissatisfied [22,23]. Moreover, 6% of women said they received conflicting advice, and of the 54% of women who had bought OTC medications in a pharmacy, only 11% were asked if they were breastfeeding by the pharmacist or pharmacy assistant [22,23]. This study, however, involved women with a high socioeconomic status and education level who were likely to breastfeed for longer, and their responses may not represent the wider population [22,23]. Further, the survey instrument developed in this study did not have the option of "Don't know" or "Can't remember" in order to counteract potential memory recall bias [22]. Similarly, in the study by Merlob, Stahl and Kaplan, only 9% of women interviewed on discharge from the maternity ward were counselled by the hospital/private pharmacist and always at woman's initiative; a lesser proportion (8%) had read the information leaflet accompanying the medication [25].

This is concerning, as pharmacists are responsible for providing accurate medication-related advice and information to the community, and it is their duty of care to check, during this process, if a woman is breastfeeding or pregnant if she is of childbearing age. It is also unsafe for pharmacists and other health professionals to assume that breastfeeding women are not likely to consider medication use, given that Schirm et al found that 66% of all breastfeeding women had used medications; albeit less frequently than non-breastfeeding women (80%) [27].

### Resources on medication safety in breastfeeding

In the only study that has been conducted to assess resources on medication safety in breastfeeding [16] (Table 3), ten different resources available to health professionals in the USA were investigated (Table 4) to evaluate 14 medications used to treat conditions such as bacterial and viral infections, hypertension, Type 2 diabetes mellitus and depression. Certainly, such resources are widely used by physicians, but less so by specialists such as endocrinologists, who prefer to conduct literature searches when unsure of the compatibility of a medicine with breastfeeding [30].

**Table 4:** List of resources, type and utility (in alphabetical order) on medication safety in breastfeeding that have been previously investigated by Akus and Bartick [16]

Name of resource	Type of resource	Utility of resource
American Academy of Pediatrics (AAP) list	Online http://pediatrics.aappublications.org/content/108/3/776.full.html	This list is reviewed by an expert panel who applies a descriptive rating system. Possible effects of agents (medicines and other treatments or products e.g. silicone implants, nicotine) on the infant or on lactation are described in tabular format. The intention is to help physicians with counselling a nursing mother regarding breastfeeding when the mother has a condition for which a medicine is indicated.
Clin-eguide http://www.ovid.com/site/products/tools/ovid/ClinicalResource.jsp	Patient handout from pharmacy database	*Clin-eguide *is an Ovid Clinical Decision Support tool for physicians. In this study, it was used by a retail pharmacy to provide information on the use of medicines in breastfeeding in the form of patient handouts (MedFacts).
Drugs in Pregnancy and Lactation	Book	Consists of monographs that include primary research and the *AAP *rating, as well as its own descriptive rating system.
Epocrates	Web-based http://www.epocrates.com/ and handheld	Program used by pharmacists and prescribers that provides a brief recommendation for each medicine, such as 'safe' or 'unknown' without additional information. The source of the recommendation is not given.
First DataBank http://www.firstdatabank.com/	Patient handout from pharmacy database	Similar to *Clin-eguide*, this is a clinical decision support tool that provides access to drug databases. In this study, it was used by a retail pharmacy to provide information on the use of medicines in breastfeeding in the form of patient handouts.
LactMed	Online http://toxnet.nlm.nih.gov/cgi-bin/sis/htmlgen?LACT	Free online resource from the National Library of Medicine that has been assembled by an expert panel. Features drug monographs and typically includes primary research, the *AAP *rating, and a summary at the top of each monograph. Unlike the *AAP list *and *Drugs in Pregnancy and Lactation*, it does not have a descriptive rating system. Among the data included are maternal and infant levels of drugs, possible effects on breastfed infants and on lactation, and alternate medicines to consider.
Lexi-Comp	Web-based http://www.lexi.com/ and handheld	Program used by pharmacists and prescribers (e.g. physicians, advanced practice nurses, dentists) that gives brief recommendations on lactation and breastfeeding considerations which are sometimes not consistent with each other. It often cites the *AAP *list but does not typically elaborate on its conclusions otherwise.
Medication and Mothers Milk	Book	Written by a clinical pharmacologist (Hale, Thomas) and updated biannually, this book features monographs on a large range of medicines (e.g. vitamins, herbs, vaccines) and environmental substances (e.g. radioisotopes, radiocontrast agents). Relevant pharmacological characteristics, primary research, and the *AAP *ratings are included in the monographs. Rather than a descriptive rating system, it applies a 5 point numerical rating system to each medicine, where L1 is safest and L5 is contraindicated.
Micromedex	Online http://www.micromedex.com/ and handheld	Used by pharmacists and other clinicians and similar to *Clin e-guide *and *First Databank*, this is a clinical decision support tool that consists of monographs that include primary research and the *AAP *rating. It also features its own descriptive rating system.
Physician's Desk Reference http://www.pdr.net/	Pharmacy database	Like *Clin e-guide *and *First Databank*, this program can be used to generate package inserts or patient handouts on the use of medicines in breastfeeding. It gives little information on how its recommendations were reached.

The advantages and disadvantages of each resource were described by Akus and Bartick, who concluded that recommendations on the safety of all of the medications in breastfeeding varied considerably, and were not based on the most recent research [16]. Two of the sources appraised were databases used in two community pharmacy chains - *Lexi-Comp *and the *Physician's Desk Reference *- that gave an alternative recommendation for medications thought to be safe at least 75% of the time [16]. Furthermore, these databases usually recommended early cessation of breastfeeding.

Certainly, the lack of, and inconsistency between, such information, could be an underlying and determining factor for the poor knowledge and variable practices displayed by health professionals towards medication use in breastfeeding women.

## Conclusions

Even though all the studies reported that there is a lack of information on medication safety in breastfeeding for health professionals [28], and the information available is inconsistent [16,17,26], in the Victorian study, most GPs were confident about medication use in breastfeeding women [18], and in the Rhode Island study, most pharmacists felt comfortable advising breastfeeding women [26]. This mismatch between available information, and comfort and confidence, needs to be further explored in future research. It is possible that some health professionals who have personally experienced breastfeeding have better knowledge and therefore comfort and confidence, leading to more appropriate decisions regarding breastfeeding and medication issues. However, "personal experiences cannot necessarily be generalised to patients and should not be relied upon" [1] (p. 28).

The lack of a uniformly accepted source of information leads to conflicting advice which can have harmful consequences for women [16]. This can also result in public distrust, discontent [22], and loss of faith towards health professionals, particularly pharmacists, who are responsible for providing medication advice, and may ultimately lead to breastfeeding women seeking advice from people who are inappropriately qualified.

Further research is required to determine whether this situation is currently occurring, as well as to scope, to a greater extent, what the educational needs of women from a range of ethnic and socioeconomic backgrounds are with respect to medication use in breastfeeding. In particular, in-depth studies are required to understand the views of women who are considering taking medicines which are regarded as risky, such as antidepressants [19,29]. Similarly, women's views about the use of other substances by breastfeeding women, such as tobacco, alcohol and caffeine, as well as illicit drugs, needs to be looked into.

At this stage however, as a result of the inconsistent information available for all stakeholders, pharmacists and other health professionals seem to be relying on their own personal experience (or a lack of it), resulting in variable practices that may mean recommending the cessation of breastfeeding and thus being overly cautious. Consequently, the infant would be deprived of the benefits that breast milk confers on growth, development, health and nutrition. Women who stop breastfeeding may perceive themselves as not being a 'good mother' which can negatively affect their self-esteem.

However, even if consistent and better quality information did become available, health professionals need to be rigorously trained on communicating with breastfeeding women via the identification, through further educational research, of continuing education formats they prefer e.g. didactic or flexible delivery methods such as online education programs. Indeed, in the study by Jones and Brown, GPs and pharmacists indicated that they would prefer a distance learning package on this topic, whereas pharmacists were more enthusiastic about interprofessional workshops [22]; however, this work was done 10 years ago. More recently, Long and Montouris reported that physicians preferred web-based or didactic material [31], but a more in-depth investigation in to health professionals' current educational needs in this area is required.

Health professionals also need to actively seek information about the breastfeeding status of women, then consider the risk of damage from the medications and the effects of the illness itself, as well as the risks of not breastfeeding, on both the woman and the baby prior to prescribing. Finally, health professionals have an obligation to extensively and adequately inform breastfeeding women about the safety of medications and their possible side effects, and to provide alternative options in circumstances where medications cannot be used, or if the mother chooses not to initiate therapy, so that more women are satisfied with the advice given to them.

The limited literature available indicates that health professionals have poor knowledge, as well as positive attitudes and variable practices that are mostly guided by personal experience, towards medication use in breastfeeding women. In turn, women are left dissatisfied with the advice provided by health professionals. Further research is needed to investigate this phenomenon. Resources available for health professionals on medication safety in lactation also need to be reviewed and updated to remove inconsistencies and reflect recent evidence as well as the experiences of expert practitioners in this area.

## List of abbreviations

GPs: General practitioners; OTC: Over-the-counter; PTU: Propylthiouracil; UK: United Kingdom; UNICEF: United Nations International Children's Emergency Fund; USA: United States of America; RR: Response rate; RWH: Royal Women's Hospital; WHO: World Health Organization

## Competing interests

The authors declare that they have no competing interests.

## Authors' contributions

SH conceived the research question to be addressed via this literature review, for the purposes of an undergraduate elective that was offered in the Bachelor of Pharmacy course at Monash University, Australia during July-October 2010. ND, an undergraduate student at Monash University, conducted the literature review, then drafted a report that was submitted for assessment by SH and the unit coordinator of the research elective. SH acted as ND's supervisor for the research elective and prepared the manuscript based on the report she produced. Both authors have read and approved the final manuscript.

## Authors' information

SH graduated from Monash University (BPharm(Hons) in 2001 and PhD in 2007) and was appointed to the position of Lecturer in 2009 at the Faculty of Pharmacy and Pharmaceutical Sciences. She is an Early Career Researcher in Pharmacy Practice with research experience and expertise in professional practice, education and medication safety and use, particularly in the area of palliative care service delivery in the primary care setting.

SH also has a strong research interest in women's health, and has recently worked on a collaborative project investigating the knowledge and attitudes of women, and the knowledge and practices of pharmacists, towards the emergency contraceptive pill that is available from pharmacies in Australia without prescription. She is currently involved in a project that will result in the production of weight management guidelines specific to women for pharmacist use in Australia, and looks forward to designing a project that will investigate gaps in Australian pharmacists', GPs' and women's knowledge, attitudes and practices towards medication use and safety in lactation.
